# Early detection of type 2 diabetes mellitus using machine learning-based prediction models

**DOI:** 10.1038/s41598-020-68771-z

**Published:** 2020-07-20

**Authors:** Leon Kopitar, Primoz Kocbek, Leona Cilar, Aziz Sheikh, Gregor Stiglic

**Affiliations:** 1 Faculty of Mathematics, Natural Sciences and Information Technologies, University of Primorska, 6000 Koper, Slovenia; 2 Faculty of Health Sciences, University of Maribor, 2000 Maribor, Slovenia; 3 Faculty of Electrical Engineering and Computer Science, University of Maribor, 2000 Maribor, Slovenia; 4 Centre for Medical Informatics, Usher Institute of Population Health Sciences and Informatics, University of Edinburgh, Edinburgh, Scotland EH8 9AG UK; 5 Division of General Internal Medicine and Primary Care, Brigham and Women’s Hospital/Harvard Medical School, Boston, MA 02115 USA

**Keywords:** Preventive medicine, Risk factors

## Abstract

Most screening tests for T2DM in use today were developed using multivariate regression methods that are often further simplified to allow transformation into a scoring formula. The increasing volume of electronically collected data opened the opportunity to develop more complex, accurate prediction models that can be continuously updated using machine learning approaches. This study compares machine learning-based prediction models (i.e. Glmnet, RF, XGBoost, LightGBM) to commonly used regression models for prediction of undiagnosed T2DM. The performance in prediction of fasting plasma glucose level was measured using 100 bootstrap iterations in different subsets of data simulating new incoming data in 6-month batches. With 6 months of data available, simple regression model performed with the lowest average RMSE of 0.838, followed by RF (0.842), LightGBM (0.846), Glmnet (0.859) and XGBoost (0.881). When more data were added, Glmnet improved with the highest rate (+ 3.4%). The highest level of variable selection stability over time was observed with LightGBM models. Our results show no clinically relevant improvement when more sophisticated prediction models were used. Since higher stability of selected variables over time contributes to simpler interpretation of the models, interpretability and model calibration should also be considered in development of clinical prediction models.

## Introduction

Type 2 diabetes mellitus (T2DM) is very common and is responsible for very considerable morbidity, mortality. Furthermore, it is a substantial financial drain both on individuals/families, health systems and societies. Of major concern is that the incidence and prevalence of T2DM are increasing rapidly—globally. In 2017, it was estimated that 425 million people had any type of diabetes (approx. 5.5% of worldwide population) of which 90% had T2DM and according to projection estimations the prevalence is going to increase substantially in the coming years; by 2,045, for example, a 48% increase of prevalence from the above numbers is expected or in absolute numbers an estimated 629 million people (approx. 6.6% of the worldwide population) are expected to be suffering from any type of diabetes^1^. T2DM can lead to substantially increased risk of macrovascular and microvascular disease, especially in those with inadequate glycaemic control^2^. Progression of T2DM from impaired fasting glucose is typically slow and more importantly, its symptoms may remain undetected for many years. Delays in diagnosis are an important contributory factor to poor control and risk of complications^3^.

Data mining is nowadays applied to various fields of science, including healthcare and medicine. Often applied are pattern recognition, disease prediction and classification using various data mining techniques^4^. Due to the increased prevalence of T2DM, various techniques have been used to build predictive models and models for early disease diagnosis, such as logistic and Cox proportional hazard regression models^5–7^, Random Forest^8,9^, boosted ensembles^10,11^, etc. The study by Damen et al.^12^ showed that logistic regression was used in most (n = 363) models for risk estimation in the general population. Even though there are multiple techniques available to build prediction models, prediction accuracy and data validity are often not realistic for model application in practice. Models also perform well in specific dataset where they were developed but are frequently not able to adapt sufficiently well with used in other datasets^5^.

Screening tools have been developed to identify individuals at high risk of developing T2DM with a view to modifying their risk factors through lifestyle modification and/or drugs. Traditional screening approaches to identify patients with undiagnosed T2DM are based on standard regression techniques. It is important to investigate whether using machine learning-based approaches can yield superior results to the currently employed methods. Specifically, conventional logistic regression is still predominantly used for development of screening tools^12^. There have however been a number of important developments in relation to machine learning methods in recent years that can now be tested for predictive modelling through interrogation of electronic healthcare record (EHR) data; these techniques include AdaBoost, random forest, support vector regression, decision tree^13^ and neural network-based on Stacked Denoising Autoencoders (SDA)^14^.

Our choice of prediction models was based on the three conceptually different families of prediction models: boosting, bagging and linear regression. This approach contributes to the credibility of the research since it reveals potentially hidden patterns that would remain hidden in the case where all applied methods used conceptually similar approach.

The aim of this study was to investigate whether novel machine learning-based approaches offered any advantages over standard regression techniques in early prediction of impaired fasting glucose (IFG) and fasting plasma glucose level (FPGL) values. Additionally, we were interested in the impact that continuous streams of new data, as is the case with EHRs, brings to the performance of prediction models. The performance of the model was not measured only using the prediction performance metrics such as AUC or AUPRC, but also by assessing the stability of selected variables over time. In the case of high variable selection stability we might get an insight in confidence of the model interpretability which could be of great help in decision on which prediction model to choose. Therefore, we simulated the incoming data in 6-month intervals and continuously compared machine learning-based prediction models with the traditionally employed regression models. In this study we hypothesized that when the new data becomes available in the EHR system it not only improves prediction performance, but also the stability of the variable importance ranking, although not equally in different machine learning prediction models.

## Methods

All methods were performed in accordance with the relevant guidelines and regulations. Due to the prior anonymisation of the data, this study belongs to the low risk records based research, meaning the informed consent by the patients was not needed^15^ as declared by the Ethical Commission at the University of Maribor Faculty of Health Sciences (approval reference number 038/2018/1779-3/501).

### Study design and research data

We undertook a retrospective study of predictive models’ derivation and validation using EHR data collected at preventive healthcare examinations of healthy population in 10 Slovenian primary healthcare institutions. Anonymisation of data was performed at the site of data collection and later pooled into a single database.

### Study setting and sample

The initial dataset comprised of EHRs from 27,050 adult individuals with no prior diagnosis of T2DM collected between December 2014 and September 2017. We removed cases and variables containing over 50% of missing values. The details of the variable and case removal process are provided in the following section on predictor variables.

### Predictor variables

Initially, the dataset consisted of 111 variables including a group of variables related to the FINDRISC (FR) questionnaire^16^ such as variables representing physical activity (at least 30 min during the day), fruit and vegetable consumption as well as keeping a track of medical history including the history of antihypertensive drug treatment, history of high blood glucose levels and family history of diabetes. Consequently, all cases with any missing FR variable were removed from the dataset to allow comparison to the FR-based model that was developed for Slovenian population by Stiglic et al.^17^.

In the next step, outliers were detected and marked as missing values, where measurements that were outside of the ${\overline{X}}\pm$(3 $\times$ SD), on the assumption of a normal distribution, were defined as outliers. The dataset was then pre-processed by removing variables and cases with 50% or more missing values. In addition to FR variables, the reduced dataset included variables that could be grouped in the following four groups: lipid profile lab results (HDL, LDL, total cholesterol and triglycerides), social determinants of health (consumption of alcohol, smoking, dietary habits, stress), cardiovascular variables (blood pressure measurements, atrial fibrillation history) and history of other health conditions (stroke, hypertension, colon cancer).

In the final step, five different subsets of data were extracted based on the time when they were collected—i.e. first 6, 12, 18, 24 and 30 months, hereafter referred to as T6, T12, T18, T24 and T30. At this stage, the information on the date of the examination was removed from all five datasets. All missing values in each of the five datasets were imputed using the Multiple Imputation by Chained Equations (MICE) missing data imputation method^18^. More specifically, missing values of numerical variables were imputed by a Bayesian linear regression method, while logistic regression was used in case of binary or dichotomous variables and polytomous regression was used in case of factor variables with more than 2 levels^18^. Each method was performed in 20 iterations, which was previously shown to be a sufficient number of iterations for an effective imputation^18^.

### Outcome

The key outcome of this study was a prediction of the current FPGL value (regression problem) based on physiological and other variables representing answers from the preventive healthcare check-up examinations. A cut-off of 6.1 mmol/L (FPGL used to determine IFG in Slovenia) was used as a threshold for the use of additional classification metrics that were used for detailed comparison of performance for different prediction models (classification problem). Application of a cut-off value resulted in a slightly unbalanced diagnostic problem in each of final subsets (Table 1).

**Table 1:** Summary information for participants with normal fasting glucose (NFG) and impaired fasting glucose (IFG) used in the study for each period separately.

PERIOD	IFG FPGL $\ge$ 6.1 mmol/L (n = 1049, 28.2%)	NFG FPGL < 6.1 mmol/L (n = 2674, 71.8%)	Number of samples (n)
T6	257 (28.9%)	635 (72.2%)	892
T12	426 (26.4%)	1185 (73.6%)	1611
T18	634 (26.8%)	1735 (73.2%)	2369
T24	798 (27.4%)	2117 (72.6%)	2915
T30	1020 (28.5%)	2560 (71.5%)	3580

### Statistical analysis and model validation

Prediction models were built and validated on each of the five final subsets separately to simulate new incoming data. Validation was conducted using 100 bootstrap runs to estimate the variability in the results. In each bootstrap iteration, a different set of samples was selected using random sampling with replacement where unselected samples were used to test the prediction models.

The following five prediction models were compared: linear regression model (lm), regularised generalised linear model (Glmnet) with Least Absolute Shrinkage and Selection Operator (Lasso) regression (L1)^19,20^, Random Forests (RF)^21^, eXtreme Gradient Boosting (XGBoost) with tree booster which uses regression tree as a weak learner^22^ and Light Gradient Boosting Machine (LightGBM) with objective set as L1 loss regression^23^. All methods were performed in accordance with the relevant guidelines and regulations.

Glmnet and lm are both linear regression methods where regularisation is used to prevent overfitting of models in Glmnet^20^. Glmnet offers different approaches to handle this problem: L1 regularisation (Lasso regression), L2 regularisation (Ridge regression) and Elastic-Net (a combination of Lasso-Ridge) penalty^19^. In this study, we utilised Lasso method, which should ensure better performance in datasets with highly correlated and sparse predictor variables, but on the other hand it can result in higher instability of the selected variables. Glmnet and lm tackle the least-squares problem in different ways. Throughout a regularisation path Glmnet applies cyclical coordinate descent algorithm in order to solve the penalised weighted least-squares problem of finding the local minimum. It works in a way that optimises the objective function for each parameter. The algorithm repeats the optimisation until convergence is achieved^19^. On the other hand, lm solves the problem of finding the local minimum by applying QR decomposition^24^ that is often used to solve the linear least squares problem.

LightGBM and XGBoost are ensemble methods based on Gradient Boosting Decision Tree (GBDT) or alternatively Gradient Boosting Machine (GBM)^22,23^. Gradient boosting is a technique where new models are added to correct the errors made by existing models—in this case, regression trees. Models improve the accuracy by fitting negative gradients, named also as residual errors^23^, which in regression symbolises a difference between expected and predicted value. XGBoost is known for its scalability in all settings, support for sparse data representation and provides higher computational speed and lower memory consumption than most other methods. On other hand, LightGBM tries to achieve similar functionality by employing two techniques called Gradient-based One-Side Sampling (GOSS) and Exclusive Feature Bundling (EFB). It is known that samples whose absolute value of gradients is larger, deliver lower training error and hence contribute to information gain more than samples whose absolute value of gradients is small. The first technique (GOSS) reduces the number of samples with keeping all instances whose absolute value of gradient is large and randomly sampling instances whose absolute values of gradients are small. Meanwhile EFB technique reduces the number of variables. Using GOSS and EFB, LightGBM profits in lower memory consumption and computational speed in comparison to XGBoost^23^.

In contrast, RF is an ensemble method based on the bagging technique. In bagging, decision trees are constructed independently. A feature of RF is that at the decision tree level, each node is divided with the best variable in a random subset of variables. This step injects some randomness to the overall model. The final result is then derived from majority voting (classification) or averaging (regression) results of all trees in RF^25^.

It is important to note that we used lm as a baseline model with a fixed set of variables. More specifically, lm was always built using only seven FR variables representing different questions, which were previously used in development of a simplified screening tool for undiagnosed T2DM and IFG in the Slovenian population^17^.

Predictive models were validated using the following performance metrics: root mean square error (RMSE) for prediction of numerical value of FPG level and AUC (area under the receiver-operating characteristic curve), AUPRC (area under the precision-recall curve) for prediction of unbalanced discrete outcome (positive and negative class).

Both prediction metrics (AUC and AUPRC) are suitable choices for model evaluation when we deal with imbalanced datasets. AUC represents a probability that a randomly selected positive instance is ranked higher than a randomly selected negative instance. AUPRC focuses on positive class and is of high importance in healthcare where positive class can represent a relatively small fraction of the population. In our study, the positive class represents 27.6% of all instances.. Since the focus in detecting IFG or impaired glucose tolerance (IGT) is on positive class we also used sensitivity (true positive rate) and positive predictive value as a performance metric for dichotomous output values. In addition, a percentage of positive predictive values was also observed as an alternative validation metric to assess the performance of different models from the economic perspective as it represents the rate of participants sent for further testing based on their screening results. Models’ performance differences (in AUC and AUPRC) were quantified with the method developed by Delong et al.^26^.

#### Variable importance

To compare the stability of the results over all five datasets, we measured variable importance for each of 58 predictor variables and each of the five prediction methods. Variable importance measures used to rank variables for each prediction model are summarised in Table 2.

**Table 2:** Description of methods used for calculating variable importance.

Prediction model	Variable importance method
lm	Variables were ranked according to the absolute value of $\beta$-coefficients. Only variables that were statistically significant were selected to build a final model in each bootstrap iteration
RF	Variables were ranked according to the increase in mean squared error (MSE), more precisely, the percentage of increase in MSE was calculated ($\Delta$MSE). For each predictor variable this method calculates the difference between MSE of Out-of-bag (OOB) data and the predicted MSE after each of predictor variable is permuted. The average difference over all trees is then normalised by standard deviation (SD) and provided as a result ($\Delta$MSE). A higher value represents a higher importance of a corresponding variable
XGBoost	Variables were ranked based on the average gain in 100 bootstrap iterations where gain represents a contribution in accuracy brought by a corresponding variable to a model
Glmnet	Fitted coefficients of variables were first standardised (i.e. each coefficient was multiplied by the standard deviation of the variable) and then ranked according to the coefficients value
LightGBM	Variables were ranked on the basis of the average gain over 100 bootstrap iterations where gain is a variable contribution to the model, measured by a variance after splitting. In LightGBM, the split point is determined by the estimated variance gain which is applied over a smaller subset. A study by Ke et al. provides more detailed information on calculating an estimated variance gain

### Ethics approval and consent to participate

The study was approved by the Ethical Commission at the University of Maribor Faculty of Health sciences with the reference number 038/2018/1779-3/501. As the data was anonymized already at the healthcare centers and due to the nature of the data needed for this study (routinely collected data) no consent from the participants was needed. Results were reported following the ‘Transparent Reporting of a Multivariable Prediction Model for Individual Prognosis or Diagnosis’ (TRIPOD)^45^ and ‘Guidelines for Developing and Reporting Machine Learning Predictive Models in Biomedical Research’^46^ statements.

### Consent for publication

None required.

## Results

### Data pre-processing

**Figure 1:**
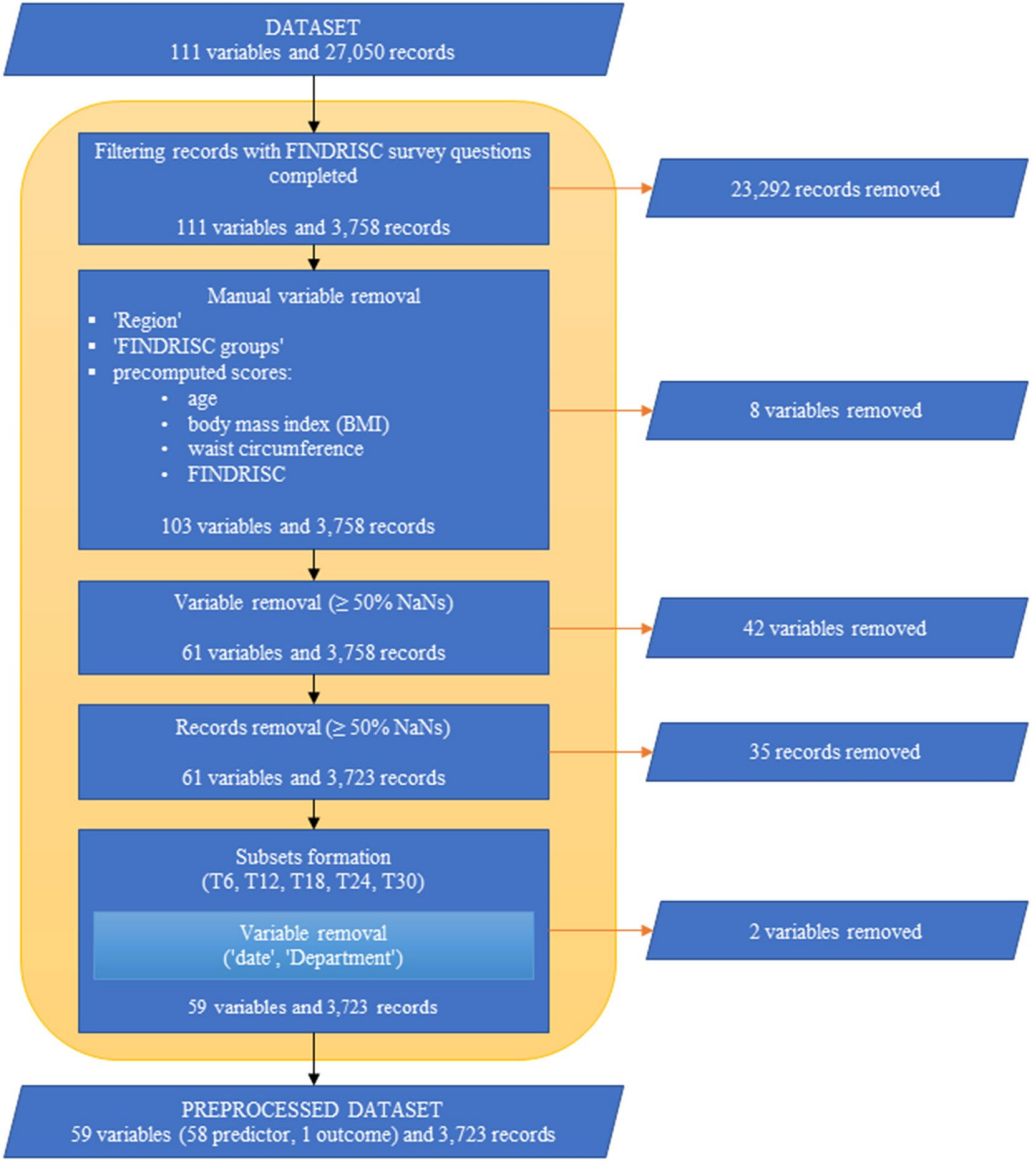
Flow diagram of data pre-processing.

Routinely collected data from EHR in ten healthcare centers in Slovenia was used in this study. The flow diagram of data pre-processing can be used as a reference while reading through this paragraph (Fig. 1). Out of a total of 27,050 patients, 3,758 patients had all FR survey questions completed. After manually removing variables like ‘Region’, ‘Finnish Diabetes Risk Score (FINDRISC) groups’ and various precomputed scores [for age, body mass index (BMI), waist circumference, FINDRISC], we reduced the number of variables from 111 to 103. For each variable, outlier values were marked as missing. Additionally, with exclusion of all variables whose proportion of missing values was above 50%, we reduced the number of variables to 61. Similar filtering was also performed on individual records level. The majority (3,723) of 3,758 records complied with the rule of less than 50% missing values. At this stage, 89.4% (3330) of records consisted of at least one missing value. Variability of missing data across different time periods prior to data imputation was minimal (T6: 9.95%, T12: 9.36%, T18: 9.04%, T24: 9.00%, T30: 8.96%).

After pre-processing, the experimental data consisted of 3,723 participants with a mean age of $54.45 \pm 11.69$ years and 61 variables, including date, department and FPGL level that were not used in model derivation. More specific information on the data can be found in the summary table (Table 1). Expectedly, patients whose FPGL was equal or higher than 6.1 mmol/L, were on average older, heavier and had larger waist circumference compared to those with normal FPGL. The IFG group included 40.2% women in comparison to NFG group with 59.2% women. This confirms our findings from earlier study in Slovenian population where we proposed stricter threshold values for screening in male populations^27^.

To simulate the new incoming data, we formed different subsets (T6, T12, T18, T24, T30), we eliminated the variable ‘Date’ in each of them and carried out imputations. Finally, prediction models [Glmnet, LightGBM, XGBoost and Random Forest (RF)] were trained and tested on the final 58 variables.

### Model performance

Initially, at T6, linear regression model (lm) performed with the lowest average Root mean square error (RMSE) of 0.838 (95% CI 0.814–0.862), followed by RF at 0.842 (95% CI 0.818–0.866), LightGBM at 0.846 (95% CI 0.821–0.871), Glmnet at 0.859 (95% CI 0.834–0.884) and XGBoost with the highest RMSE of 0.881 (95% CI 0.856–0.907). When more samples were added, every single model showed improvement. Considering the time period before each addition of new data, Glmnet ($T6_{RMSE}$ = 0.859) improved at the highest rate (+ 3.43%) with an average decrease in RMSE of $- 0.040 \pm 0.017,$ followed by XGBoost ($T6_{RMSE}$ = 0.881) having an average decrease of $- 0.028 \pm 0.018$ (+ 3.2%). On the other hand, lm’s performance ($T6_{RMSE}$ = 0.838) improved at the slowest rate (+ 2.7%) with an average decrease in RMSE of $-0.016 \pm 0.015$ over the observed period (T6–T30). At the final time point with 30 months of available data, RF performed with the lowest average RMSE of 0.745 (95% CI 0.733–0.757), just slightly lower than Glmnet with 0.747 (95% CI 0.734–0.759), where XGBoost showed the highest RMSE of 0.760 (95% CI 0.748–0.772). XGBoost performed with the highest average RMSE among all models for all five datasets. However, based on the observed trend in RMSE it would be possible for XGBoost to perform better than other models when more data would be available.

In terms of area under the receiver-operating characteristic curve (AUC) metric, Glmnet outperformed all compared methods on datasets T18–T30. The AUC of 0.818 (95% CI 0.813–0.822) on the data collected within the first 6 months was lower than the AUC of the RF model that achieved an AUC of 0.819 (95% CI 0.815–0.823). Using the T30 dataset, Glmnet maintained the best results with the AUC of 0.859 (95% CI 0.857–0.861), compared to lm with the AUC of 0.854 (95% CI 0.852–0.856), RF with 0.852 (95% CI 0.850–0.854), LightGBM with 0.847 (95% CI 0.845–0.849) and XGBoost with 0.844 (95% CI 0.842–0.846), respectively.

Similarly, lm and Glmnet showed higher value of area under the precision-recall curve (AUPRC) than other models. On average, lm marginally surpassed Glmnet in every single time period. The best performance in AUPRC was achieved at T30 where lm and Glmnet achieved an AUPRC of 0.747 (95% CI 0.743–0.751) and 0.740 (95% CI 0.736–0.744), respectively.

To assure that mentioned differences among models are significant, we decided to quantify the performance differences (in AUC and AUPRC) between all pairs of prediction models (see SI Table S1 online) using a method proposed by DeLong et al.^26^. In none of the time periods, neither of the used prediction model pairs showed a non-significant difference in both performance metrics. Initially at the T6, there are some prediction model pairs with a significant difference in only one of the mentioned metrics [(Glmnet-lm (AUC), RF-lm (AUC), Glmnet-RF (AUC), LightGBM-RF (AUPRC), LightGBM-XGBoost (AUPRC)]. After the first addition of the new samples (T12) some model pairs became significantly different in both metrics (RF-lm, Glmnet-RF, LightGBM-XGBoost). In the latest time period (T30), significant differences were noticed between all prediction model pairs except LightGBM-RF (see SI Table S1 online).

Interestingly, Glmnet and XGBoost were the only models whose average sensitivity (SENS) consistently increased with each additional batch of available data. On average, the sensitivity of Glmnet and XGBoost improved by 1.2% and 5.5%, respectively. It is important to note that XGBoost was at T6 predicting with the lowest sensitivity of 0.702 (95% CI 0.694–0.710) in comparison to Glmnet ($T6_{SENS}$ = 0.729 (95% CI 0.720–0.738)) and other prediction models. On average, the highest sensitivity was achieved by Glmnet after the last addition of data (T30) with 0.764 (95% CI 0.759–0.760).

The percentage of positively predicted outcomes decreased over time when more data became available and was approaching the true percentage of positive samples.

### Variable importance

**Figure 2:**
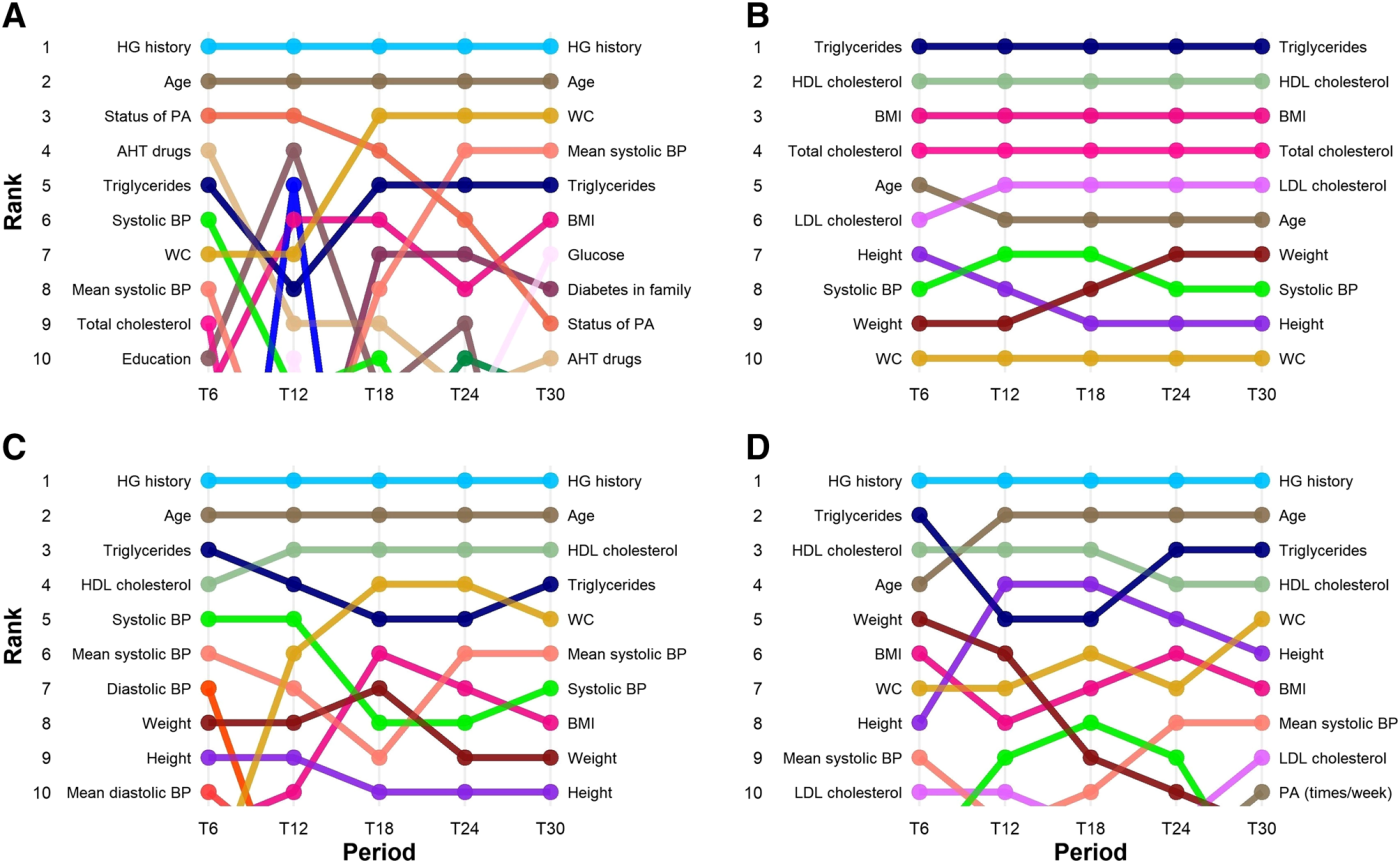
Variable importance. Ranking of variables for Glmnet (**A**), LightGBM (**B**), Random Forest (**C**) and XGBoost (**D**) over the observed period (T6–T30).

The highest variable importance score was observed for the variable of previously observed hyperglycemia (Fig. 2). It was ranked as the most important variable in three out of four prediction models over all five datasets. Interestingly, the same variable was not ranked in the top 10 positions for the fourth prediction model (LightGBM). Patient age was found as the second most important variable in three models (RF, XGBoost, Glmnet). Across all datasets (T6–T30), Level of high-density lipoprotein (HDL) cholesterol was ranked between second and fourth position for RF, XGBoost and LightGBM.

Another laboratory clinical measurement, ‘Triglycerides’ was constantly ranked in the top five positions, with exception at T12 (model Glmnet) where its rank dropped to the eight position for a short time. It was interesting to note a decrease in ranking for ‘Use of antihypertensive drugs’ and especially ‘Status of physical activity’ with increasing sample size. In general, LightGBM resulted in the most stable performance in terms of variable importance-based ranking. This finding was important as high variability of variable ranking over time complicates interpretation of derived models meaning that reinterpretation would often be needed.

### Model calibration

**Figure 3:**
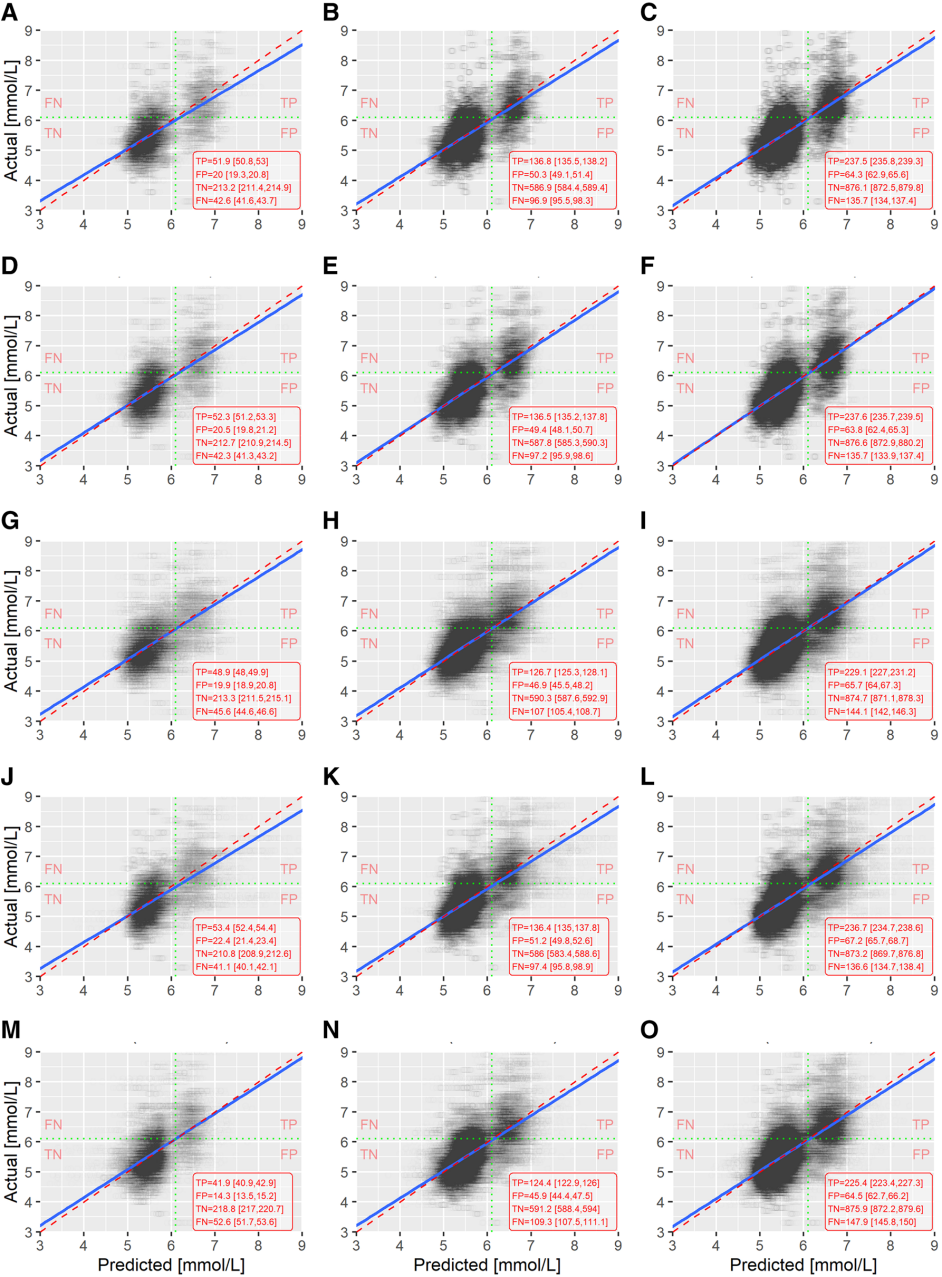
Actual vs. predicted plots. Visualisation of actual vs. predicted values for all predictive models (lm, Glmnet, LightGBM, RF, XGBoost) in three time points (T6, T18 and T30) reveal discrepancies in calibration of the compared models. Additional classification performance results in terms of TP, FP, TN and FN are provided where it can be seen that lm, Glmnet and RF outperformed both boosting based methods by identifying more TP as well as TN cases. Model-time point combinations are represented in the following way: lm 6, 18 and 30 months (**A**–**C**), Glmnet 6, 18 and 30 months (**D**–**F**), LightGBM 6, 18 and 30 months (**G**–**I**), RF 6, 18 and 30 months (**J**–**L**), XGBoost 6, 8 and 30 months (**M**–**O**).

Next, we compared model calibration and observed differences in results based on visual inspection of actual vs predicted FPGL (Fig. 3). The normal coefficients of determination ($R^2$)^28^ indicate that none of the models were close to the moderate performance as regressors. A general guideline suggests that the $R^2$ values of 0.75, 0.50 and 0.25 are considered as substantial, moderate and weak levels of predictive accuracy^29^. Looking at $R^2$, no significant differences between prediction models were found. As expected $R^2$ increased for each model with the increased sample size. Taking into an account the average $R^2$ values (T6, T18, T30), it is noticeable that the largest and steadiest improvement step was achieved by XGBoost (T6–T18: + 0.052, T18–T30: + 0.050) (Table 3). The same observation reflects in prediction models’ performance metrics AUC (Table 4), AUPRC (Table 5), as well as in metric RMSE (Table 6).

**Table 3:** Coefficients of determination ($R^2$) of prediction models at three time points (T6, T18 and T30).

$$R^2$$			
Prediction model	T6	T18	T30
lm	0.310 [0.301, 0.319]	0.326 [0.319, 0.332]	0.358 [0.351, 0.365]
Glmnet	0.281 [0.269, 0.293]	0.330 [0.322, 0.337]	0.366 [0.358, 0.373]
LightGBM	0.293 [0.284, 0.302]	0.316 [0.308, 0.323]	0.348 [0.341, 0.355]
RF	0.305 [0.297, 0.314]	0.340 [0.333, 0.348]	0.369 [0.362, 0.376]
XGBoost	0.241 [0.232, 0.249]	0.293 [0.286, 0.300]	0.343 [0.336, 0.349]

Results are shown as average values with corresponding 95% CI.

**Table 4:** Area under the curve (AUC) of prediction models at three time points (T6, T12, T18, T24 and T30).

AUC					
Prediction model	T6	T12	T18	T24	T30
lm	0.817 [0.812, 0.821]	0.813 [0.809, 0.816]	0.835 [0.833, 0.838]	0.842 [0.840, 0.844]	0.854 [0.852, 0.856]
Glmnet	0.818 [0.813, 0.822]	0.815 [0.811, 0.819]	0.841 [0.839, 0.844]	0.847 [0.845, 0.850]	0.859 [0.857, 0.861]
LightGBM	0.807 [0.803, 0.812]	0.808 [0.804, 0.811]	0.827 [0.825, 0.830]	0.837 [0.834, 0.839]	0.847 [0.845, 0.849]
RF	0.819 [0.815, 0.823]	0.810 [0.807, 0.814]	0.833 [0.831, 0.836]	0.840 [0.838, 0.843]	0.852 [0.850, 0.854]
XGBoost	0.789 [0.784, 0.794]	0.785 [0.782, 0.789]	0.820 [0.817, 0.823]	0.833 [0.831, 0.835]	0.844 [0.842, 0.846]

Results are shown as average values with corresponding 95% CI.

**Table 5:** Area under the precision-recall curve (AUPRC) of prediction models at three time points (T6, T12, T18, T24 and T30).

AUPRC					
Prediction model	T6	T12	T18	T24	T30
lm	0.671 [0.664,0.678]	0.642 [0.636,0.648]	0.697 [0.693,0.702]	0.717 [0.713,0.721]	0.747 [0.743,0.751]
Glmnet	0.658 [0.651,0.666]	0.634 [0.627,0.641]	0.696 [0.692,0.701]	0.710 [0.705,0.715]	0.740 [0.736,0.744]
LightGBM	0.641 [0.633,0.649]	0.616 [0.610,0.623]	0.673 [0.668,0.678]	0.695 [0.690,0.699]	0.723 [0.719,0.727]
RF	0.648 [0.640,0.656]	0.614 [0.607,0.621]	0.683 [0.678,0.688]	0.694 [0.690,0.699]	0.723 [0.719,0.727]
XGBoost	0.632 [0.623,0.640]	0.600 [0.594,0.607]	0.656 [0.651,0.661]	0.685 [0.680,0.689]	0.715 [0.711,0.719]

Results are shown as average values with corresponding 95% CI.

**Table 6:** Root mean square error (RMSE) of prediction models at three time points (T6, T18 and T30).

RMSE			
Prediction model	T6	T18	T30
lm	0.838 [0.814, 0.862]	0.790 [0.774, 0.806]	0.751 [0.738, 0.763]
Glmnet	0.859 [0.834, 0.884]	0.788 [0.772, 0.804]	0.747 [0.734, 0.759]
LightGBM	0.846 [0.821, 0.871]	0.796 [0.780, 0.813]	0.758 [0.745, 0.770]
RF	0.842 [0.818, 0.866]	0.782 [0.766, 0.798]	0.745 [0.733, 0.757]
XGBoost	0.881 [0.856, 0.907]	0.809 [0.793, 0.825]	0.760 [0.748, 0.772]

Results are shown as average values with corresponding 95% CI.

By observing the scatter plots we noticed that in the two most basic models (i.e. lm and Glmnet) the samples around the FPGL of 6.1 mmol/L were most clearly separated, thus separating the normal blood glucose level and IFG participants in two groups.

In addition, we noticed that fasting glucose levels predicted by the XGBoost on the data collected in the first six months rarely crossed the threshold of 7.0 mmol/L. Consequently, such a predictive model would be of very limited use if applied to the undiagnosed T2DM prediction.

## Discussion

We compared performance, calibration and interpretability of machine learning-based prediction models to multivariable regression models when predicting FPGL and presence of T2DM. Machine learning methods in combination with other concepts introduced in the learning healthcare systems approach have a potential to deliver better care and management of T2DM to health care providers, service users and lay people. However, when introducing novel prediction models, one should take into consideration not only the predictive performance, but also calibration and interpretability of the models where the benefits and drawbacks of the machine learning methods need to be taken into consideration.

Different data mining approaches were used in studies to predict T2DM, diabetic complications, genetic background, health care and management of T2DM^30^. Similar methods have also been used in prognosis and prediction of other diseases, such as cancer^31–33^ and cardiovascular diseases^34,35^. However, it is always difficult to select the most appropriate machine learning methods for a specific problem one is trying to solve. In this study, we therefore selected ensemble-based methods that were recently used in similar studies and demonstrated the best results, especially in terms of performance. The pool of available machine learning methods is too wide to test all or the majority of them. Additionally, each of the machine learning-based approaches can be tuned by changing the values of parameters needed to build a predictive model and improve its performance. So even with a limited number of machine learning models included in a study there is practically an infinite number of possible parameter combinations. Therefore, we aimed to set the parameters in a way where the computational complexity and performance would be as balanced as possible. Consequently, it is also very important to plan a robust validation strategy where training and testing set are separated also in the parameter tuning process which additionally increases the time complexity.

A limitation of this study is that we only used one database with a limited number of available variables and a large amount of missing data. Another limitation relates to the population studied. As the participants at the preventive examinations consisted of predominantly working population, the dataset did not include older people in whom T2DM is more prevalent. On the other hand, the working population represents the most appropriate population for early interventions in lifestyle to avoid later complications.

In one of the recent studies, Christodoulou et al. conducted a systematic review where in 71 studies the performance of machine learning models did not significantly surpass the performance of logistic regression^36^. Similarly, our study shows no significant improvement when using sophisticated prediction models. Similar studies have included different variables in prediction models that were adjusted to the characteristics of specific population. Our results show that highest ranked variables in prediction of T2DM include hyperglycemia history, age, HDL cholesterol, triglycerides, physical activity and antihypertensive drugs (Fig. 2). Both variables, history of high blood glucose levels (HG History) and age (Age) are present in most screening tests, which was confirmed in our study for all prediction models except LightGBM. In the case of LightGBM, the most significant variables included triglycerides and blood cholesterol levels. An elevated level of triglycerides is present at 60% up to 70% of diabetic patients^37^. Additionally, a recent study by Alexopoulos et al.^38^ recommends treatment of triglycerides as an emerging target in diabetes care. However, from a clinical perspective, questions related to the laboratory results are not convenient as a part of a screening test, since they are time consuming, expensive and difficult to obtain in some environments.

Similar results were found in the previous research conducted in Slovenia^17^, where the most important variables were hyperglycemia history, gender, age, physical activity, waist circumference, (BMI), diabetes in family, fruit and vegetables consumption and antihypertensive drugs. Variables as age, parental history of diabetes and BMI were statistically significant predictors of T2DM already in The Framingham Offspring Study^39^. Recent studies synthesised in the literature review conducted in 2017 have found that main factors in developing T2DM are following: age, gender, height, BMI, waist circumcise, blood pressure, HDL cholesterol and others^40,41^. Observing the variables that were selected using machine learning techniques (Fig. 2), we can conclude that there are major differences in comparison to simpler models like multiple logistic regression or in comparison to similar studies based on predictive models in Slovenia and elsewhere.

Most of the top ranked variables are present in all four lists. However, there are variables that were not selected in a specific model, but there are usually other, so called, proxy variables that were selected instead. For example, LightGBM based models did not rank HG History in the top 10 variables which was the case in all other models. On the other hand LightGBM based model ranked all four lab results and BMI in the top 5 most influential variables which rarely happened in other models.

It is also interesting that LightGBM based models resulted in higher stability in ranking the influential variables in comparison to conceptually similar XGBoost based models as both methods follow the idea of boosting the prediction models. However, there is a technical detail that could explain the results of LightGBM which uses leaf-wise instead of level-wise decision tree growth used in XGBoost^23^. In leaf-wise tree growth the number of selected variables is smaller and the tree can be built faster. Consequently, the variability of selected variables is smaller in comparison to approaches where the complete level of nodes is expanded in parallel.

Comparison based on the increasing number of available cases to build prediction models has previously been studied by Yang et al.^42^ who compared machine learning methods on the same sample with different training sizes. Despite high variance in the results, it was demonstrated that with increased sample size, $R^2$ also increased, while RMSE decreased, indicating that the model explained more variability of the response variable and a better fit with increased sample size^42^. Similar findings were presented in a study by Johansson, et al. where prediction models that were trained on a progressively increasing training dataset performed more accurately [lower mean absolute error (MAE) and higher $R^2$] than the fixed model build on the initial set of initially available data. Furthermore, they concluded that MAE and $R^2$ are alone insufficient for determining public health utility^43^. Results from both studies are in concordance with the results obtained in this study where we showed that the sample size increased from 0.26 (T6) to 0.36 (T30) for Glmnet, from 0.28 (T6) to 0.34 (T30) for LightGBM, from 0.29 (T6) to 0.36 (T30) for RF and from 0.29 (T6) to 0.35 (T30) for lm prediction model. Furthermore, Olivera et al.^44^ suggested that the best prediction models are those created by using machine learning algorithms, such as artificial neural networks and logistic regression.

Modern modelling techniques allow us to predict many different health related outcomes. There is an obvious difference in predictive performance of such predictive models, which differ because of different datasets, techniques and methods for developing those models^44^. Our study showed that we can expect very limited performance gain when predicting undiagnosed pre-diabetes and T2DM or FPGL using machine learning-based approaches in comparison to logistic regression-based model. Similar results were obtained in a recent study by Christodoulou et al.^36^. Therefore, one should base a decision on which predictive model to choose on model calibration, interpretability and stability of results over time and not just predictive performance. Based on the results presented in this study, LightGBM is a reasonable choice when stable performance in terms of variable importance-based ranking is the most desirable characteristic. The RF model provided a balanced combination of good interpretability and performance in terms of reducing the incorrectly predicted negative levels (False Negatives). In addition, RF constantly performed well in terms of RMSE at the beginning, and all the way up to the final dataset with all available data. However, both regression-based models achieved very similar results and would still represent an optimal choice in our case, especially as they are simple to interpret and implement in practice.

## Conclusions

We studied differences in performance, calibration and interpretability of machine learning-based prediction models and multivariable regression models. Our results show that using new data in the EHR system to rebuild prediction models not only improves prediction performance, but also stability of the variable importance ranking, although not equally in different machine learning prediction models. Our results found no clinically relevant improvement when employing machine learning-based models over the more conventional regression models in terms of predictive performance. Even with calibration of the models, visualisation of the observed versus actual FPGL showed some advantages in using simpler models. When observing the stability of variable ranking based on relative importance of variables, one can notice that a method like LightGBM results in much more stable results in comparison to other methods, which were more prone to high variance in variable importance. Both regression-based methods also proved as comparable alternatives. Since regression-based prediction models have been regularly used in clinical practice they could represent a better alternative in some clinical environments. The results in this study show significant improvement in terms of AUC, AUPRC and RMSE for all tested methods as the amount of collected data increases. For all tested predictive models in most of the experiments, we were able to show that additional data availability positively correlates with improved predictive performance and more stable variable importance-based ranking of variables. The opportunity of updating models arises as additional routine data become available over time. Future research needs to explore the implementation of different approaches of building ensemble methods. In this case, stacking and blending of different prediction models could be taken into consideration. However, such systems bring along even more challenges in terms of interpreting the results that should support decisions of the healthcare experts.

## Electronic supplementary material

Supplementary material 1 (pdf 220 KB)

## Data Availability

The dataset analysed during the current study is not publicly available due to non-disclosure of microdata agreement between the data providers and the researchers but are available from the corresponding author on reasonable request and with agreement of data providers.
