# Supplementary material for: Early detection of type 2 diabetes mellitus using machine learning-based prediction models
Source: Sci Rep. 2020 Jul 20;10:11981. doi: 10.1038/s41598-020-68771-z (PMC7371679; doi:10.1038/s41598-020-68771-z)
Supplement: Supplementary file 1 — Supplementary material 1 (pdf 220 KB) [file 41598_2020_68771_MOESM1_ESM.pdf]

Supplementary information

Early detection of type 2 diabetes mellitus using machine learning-based prediction models

Leon Kopitar[1,*], Primož Kocbek[2], Leona Cilar[2], Aziz Sheikh[4,5], and Gregor Štiglic[2,3]

[1]Faculty of Mathematics, Natural Sciences and Information Technologies, University of Primorska, Koper, 6000,Slovenia

[2]Faculty of Health Sciences, University of Maribor, Maribor, 2000, Slovenia

[3]Faculty of Electrical Engineering and Computer Science, University of Maribor, Maribor, 2000, Slovenia

[4]Centre for Medical Informatics, Usher Institute of Population Health Sciences and Informatics, University ofEdinburgh, Edinburgh, EH8 9AG, Scotland, UK

[5]Division of General Internal Medicine and Primary Care, Brigham and Women's Hospital/Harvard Medical School,Boston, MA, 02115, USA

*leon.kopitar@famnit.upr.si

T6	lm	Glmnet	LightGBM	RF	XGBoost	
**lm**	1	1.71E-08	1.71E-08	9.86E-06	2.43E-12	**AUPRC**
**Glmnet**	3.35E-01	1	1.15E-03	4.27E-02	1.98E-06	
**LightGBM**	5.92E-05	4.91E-09	1	2.43E-01	1.07E-01	
**RF**	1.43E-01	3.78E-01	2.53E-20	1	6.49E-03	
**XGBoost**	1.64E-33	8.31E-44	6.55E-20	2.76E-44	1	
	**AUC**					

T12	lm	Glmnet	LightGBM	RF	XGBoost	
**lm**	1	9.37E-02	1.96E-08	6.63E-10	5.71E-20	**AUPRC**
**Glmnet**	8.70E-03	1	3.99E-04	4.85E-05	1.20E-11	
**LightGBM**	4.35E-04	5.25E-11	1	5.77E-01	7.15E-04	
**RF**	4.66E-02	6.66E-08	1.11E-02	1	4.90E-03	
**XGBoost**	4.52E-66	9.75E-98	1.76E-51	3.38E-61	1	
	**AUC**					

T18	lm	Glmnet	LightGBM	RF	XGBoost	
**lm**	1	7.35E-01	4.17E-13	1.64E-05	3.68E-31	**AUPRC**
**Glmnet**	1.76E-18	1	1.14E-11	9.21E-05	1.15E-28	
**LightGBM**	8.98E-14	1.67E-60	1	6.44E-03	3.03E-06	
**RF**	7.09E-02	1.74E-28	4.48E-20	1	7.68E-13	
**XGBoost**	1.86E-45	2.48E-109	2.01E-14	2.69E-41	1	
	**AUC**					

T24	lm	Glmnet	LightGBM	RF	XGBoost	
**lm**	1	1.95E-02	3.85E-13	5.81E-14	2.34E-26	**AUPRC**
**Glmnet**	7.33E-22	1	4.81E-06	1.82E-06	1.93E-14	
**LightGBM**	2.55E-08	1.95E-50	1	8.79E-01	2.45E-03	
**RF**	1.08E-01	5.16E-32	7.05E-10	1	3.65E-03	
**XGBoost**	4.50E-24	2.70E-83	7.92E-07	1.34E-20	1	
	**AUC**					

T30	lm	Glmnet	LightGBM	RF	XGBoost	
**lm**	1	6.26E-03	3.07E-19	8.22E-19	3.85E-33	**AUPRC**
**Glmnet**	7.79E-23	1	2.85E-09	7.51E-09	2.61E-18	
**LightGBM**	2.97E-21	6.58E-85	1	7.96E-01	8.45E-03	
**RF**	2.20E-03	6.04E-45	1.63E-22	1	3.24E-03	
**XGBoost**	2.53E-48	3.96E-133	3.09E-08	3.83E-38	1	
	**AUC**					

Supplementary Table S1: Quantification of performance differences between models (AUC and AUPRC). Each cell corresponding to a pair of prediction models contains a p-value.
